# Observed and perceived benefits of providing physical activity opportunities in elementary schools: a qualitative study

**DOI:** 10.3389/fspor.2023.1240382

**Published:** 2023-08-31

**Authors:** Timothy J. Walker, Derek W. Craig, Christopher D. Pfledderer, Michael C. Robertson, Paula Cuccaro, Keisey Fumero, John B. Bartholomew

**Affiliations:** ^1^ Department of Health Promotion and Behavioral Sciences, University of Texas Health Science Center at Houston School of Public Health, Houston, TX, United States; ^2^ Department of Health Promotion and Behavioral Sciences, University of Texas Health Science Center at Houston School of Public Health, Austin Regional Campus, Austin, TX, United States; ^3^ Department of Nutrition, Metabolism, & Rehabilitation Sciences, The University of Texas Medical Branch, Galveston, TX, United States; ^4^ Children’s Learning Institute, University of Texas Health Science Center at Houston, Houston, TX, United States; ^5^ Department of Kinesiology and Health Education, The University of Texas at Austin, Austin, TX, United States

**Keywords:** physical activity, school, academics, behavior, student, teacher

## Abstract

**Introduction:**

Schools play an important role in promoting physical activity for youth. However, school-based physical activity opportunities often compete with other academic priorities, limiting their implementation. The purpose of this study was to qualitatively explore elementary school teacher and staff perspectives on providing physical activity opportunities and how they impact students and learning.

**Methods:**

We partnered with a school district in Texas to conduct semi-structured individual interviews. We used a purposeful sampling approach to recruit elementary teachers and staff knowledgeable about the physical activity opportunities provided at their school. Interviews included questions about participant opinions of providing physical activity opportunities and the types of opportunities provided. We analyzed data using a directed content analysis and iterative categorization approach.

**Results:**

Fifteen participants (4 teachers, 4 physical education teachers, 3 assistant principals, and 4 principals) completed interviews from 10 elementary schools. Participants discussed observed and perceived benefits when providing physical activity opportunities, which emerged into four themes and subthemes: (1) academic benefits (learning readiness, learning engagement, and academic performance); (2) social-emotional benefits (behavior, interpersonal and social skills, and classroom culture); (3) physical benefits (brain health, skill development, physical health); and (4) instructional benefits (quality teaching time, helpful teaching tools, and teacher-student relationships).

**Conclusions:**

Teachers and staff observed numerous benefits when students had opportunities to be physically active, including the positive impact on academic and social-emotional outcomes. Our findings highlight the alignment of physical activity with other school priorities. Physical activity programming can be used in ways to support academics, learning, behavior, and other important outcomes.

## Introduction

The health benefits of physical activity for youth are well-documented as higher levels of physical activity are associated with better cardiorespiratory fitness, muscular fitness, bone health, and weight status (1). Physical activity is also associated with academic, learning, and social emotional outcomes. Specifically, studies have reported positive associations between physical activity and standardized test scores, math performance, academic performance, academic achievement (2–5), school readiness, attention, time-on-task, and student behavior (4, 6–10).

Given the many benefits, national and global guidelines recommend youth participate in 60 min of moderate-to-vigorous aerobic physical activity each day (11). Schools play an important role in helping youth engage in physical activity and are further recommended to provide ≥30 min of physical activity during school hours (12, 13). Traditionally, physical education and recess were the primary sources of students' school-based physical activity, although fiscal and policy pressures have led to reductions in these opportunities (13, 14). As a result, authorities recommend schools supplement physical education and recess with multiple approaches to support physical activity, including classroom-based approaches, before/afterschool programs, and active transport to and from school (13, 15, 16).

Despite the promise of multicomponent physical activity approaches (17), they remain challenging to implement, even when mandated by district or federal policy (18). Common implementation barriers include competing priorities, an unsupportive culture and a lack of time, resources, staff capacity, staff buy-in, and leadership support (19–22). For example, studies examining perspectives of those implementing multicomponent physical activity approaches have reported the importance of obtaining leadership support and how academic subjects usually take priority over physical activity initiatives (20, 23). Additionally, a recent meta-synthesis of studies on physically active learning reveals the complexity of implementation across internal (motivation, self-efficacy) and external (training, resources, school culture) factors (24). Schools operate in a resource-limited environment, where time and resources (e.g., staffing or materials) for physical activity opportunities can be in direct competition with other academic priorities. This is particularly true as schools respond to the pressures of high stakes, standardized testing by reducing physical activity opportunities to allow for more academic instruction (25, 26). As scheduling and resource allocation decisions are made at the local level, there is a need to better understand how educators view physical activity opportunities.

Given the often complex and dynamic nature of implementing physical activity opportunities in the school setting and the need to deepen our understanding of how differing priorities in schools may help or hinder physical activity promotion, it is important to provide teachers and school administrators with the space to detail their experiences with school-based physical activity. A lack of understanding of educator's perspectives about physical activity can lead to misguided efforts for how best to communicate and promote physical activity in schools. Thus, the purpose of this study was to qualitatively explore elementary school teacher and staff perspectives on providing physical activity opportunities and how they impact students and learning.

## Methods

### Setting and participants

We partnered with a school district in southeast Texas during the spring of 2018 to conduct this qualitative study. At the time of the study, the district had over 40 schools and served approximately 35,000 students annually. Over half of the district's elementary schools were designated as Title 1 (schools that have ≥40% of enrolled students who were considered economically disadvantaged and qualified for free or reduced-price lunch). Individuals who were involved with supporting physical activity opportunities and employed at any of the district's elementary schools were eligible to participate in the study. The study and all procedures were approved by the Committee for Protecting Human Subjects at The University of Texas Health Science Center at Houston (HSC-SPH-17-0980) and the school district's Research and Evaluation department.

During the time of study, the district wellness department was actively supporting physical activity opportunities in numerous ways. They encouraged schools to adhere to the state physical activity policy for physical education (also referred to as health fitness by the district) and the district recess policy (30 min/day). Wellness staff also helped facilitate partnerships for before/afterschool programs, and actively promoted the use of classroom-based approaches such as brain breaks (short physical activity sessions that provide a break from traditional didactic instruction), physically active learning (integrating movement into academic instruction), and motorlabs (designated spaces with ready-to-use equipment for physically active learning) (27).

### Procedures

We used a purposeful sampling approach to identify and select elementary school staff from across the district. First, we worked with the district wellness team to identify staff who would be willing to speak about their school's physical activity practices. Then, we emailed staff to introduce the study and scheduled interviews with those interested. As recruitment progressed, we balanced the study sample across four job types to obtain different perspectives within the school setting: (1) principals, (2) assistant principals, (3) physical education teachers, and (4) classroom teachers. We chose to focus on these job types given feedback from interviewees and our input from the district wellness team.

One member of the research team (lead author) completed all of the interviews. The interviews were semi-structured and focused on topics related to school-based physical activity. Specifically, the interview guide included questions about participant opinions on providing physical activity opportunities for students, the types of opportunities provided, and how schools implemented these opportunities. Each interview lasted between 45 and 60 min and participants were provided with a $30 gift card for their participation. Participants had the option to complete interviews at their school or at a different location (e.g., public park or appropriate public space). We chose interview locations based on participant preferences. We obtained written consent from all participants prior to completing the interviews and the transcripts were professionally transcribed verbatim. We stopped recruiting additional participants when minimal new information was generated from additional interviews and when the team felt the study goals could be achieved given the data quality (28).

### Analysis

We analyzed the data using a directed content analysis (29) and iterative categorization approach (30). Three research team members independently coded three interview transcripts and discussed each transcript to generate a codebook. The team also consulted with the qualitative project lead to attain feedback on the codebook. The team then coded the remaining 12 transcripts (lead author coded all transcripts, supporting team members each coded 6). The team met regularly during the coding process to discuss codes and establish consensus. During the coding process, the team included a general code that pertained to the benefits of physical activity. Using the excerpts from this general code, two team members conducted a focused review of all excerpts, applied specific benefits codes (e.g., a specific code for improved focus), and discussed the additional codes to reconcile discrepancies. The two team members also generated matrices (codes by interviewees) as a way to summarize and compare data. They shared the matrices with members of the research team to further group the reported benefits into emergent categories of related concepts. We used Dedoose (Version 8, SocioCultural Research Consultants, LLC, Los Angeles, CA) for coding and Microsoft Excel to complete the matrices and subsequent analysis.

## Results

**Figure 1:**
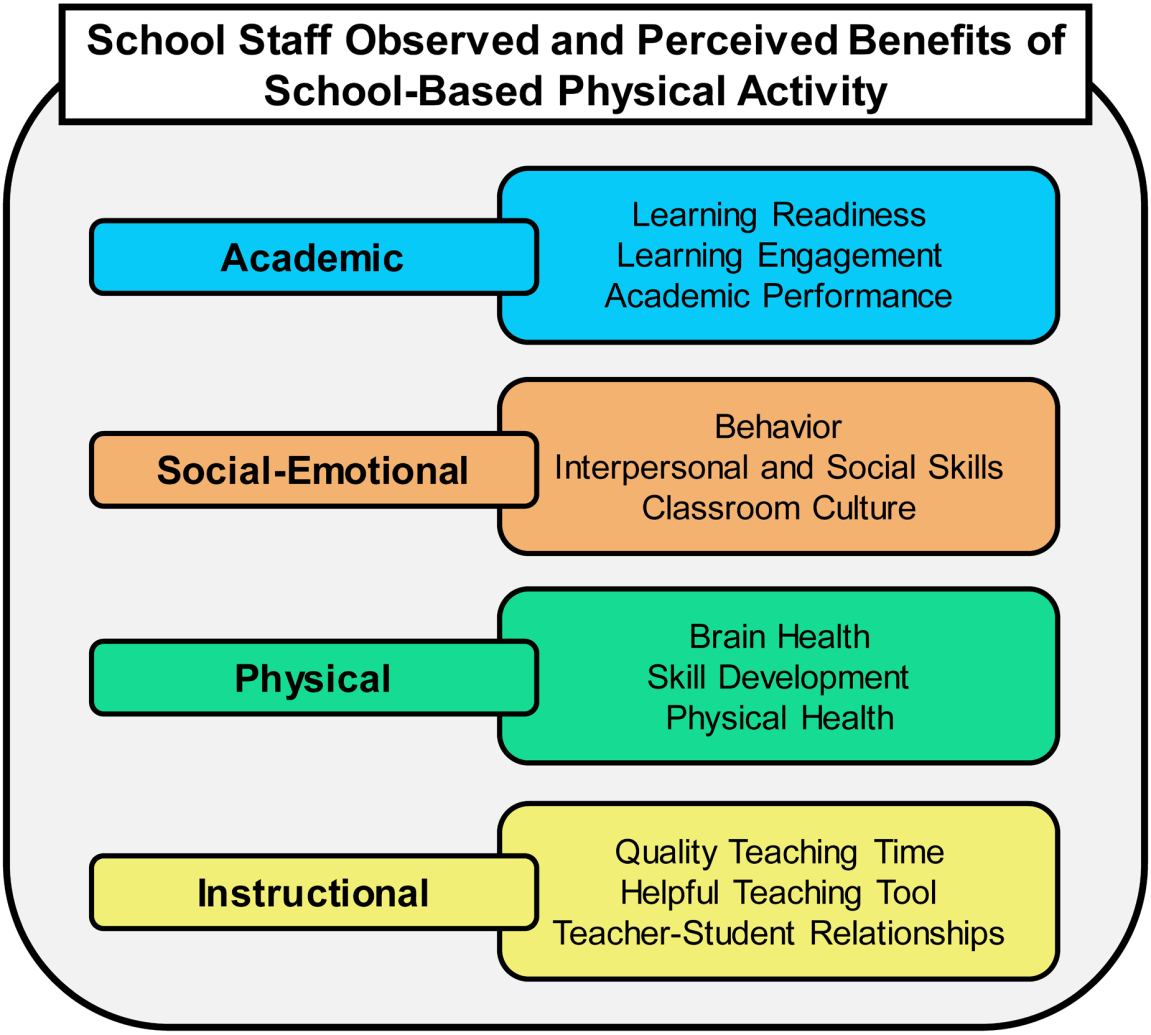
Observed and perceived benefits of school-based physical activity.

Fifteen participants (4 teachers, 4 PE teachers, 4 principals, and 3 assistant principals) completed interviews from 10 different elementary schools (Tables 1, 2). Participants discussed multiple benefits when providing physical activity opportunities for students, which emerged into four themes: (1) academic benefits, (2) social-emotional benefits, (3) physical benefits, and (4) instructional benefits. Within each theme, there were also various subthemes. Table 3 provides an overview of how staff spoke about the physical activity benefits. Figure 1 provides an overview of themes and subthemes.

**Table 1:** Characteristics of study sample.

Variable	Total sample (*n* = 15)
Female (%, *n*)	93 (14)
Age (%, *n*)	
26–35	13.3 (2)
36–45	33.3 (5)
46–55	40.0 (6)
≥56	13.3 (3)
Years in current position (m, SD)	8.5 (6)

**Table 2:** Characteristics of schools.

Variable	Total sample (*n* = 10)
Title 1 (%, *n*)	90 (9)
Average percentage of students who are economically disadvantaged	70.0 (26.9)
School race/ethnicity (%, *n*)	
Majority white (≥50% of students served are non-hispanic white)	20 (2)
Majority hispanic (≥50% of students served are hispanic)	70 (7)
Diverse (no single race/ethnicity group ≥50%)	10 (1)
Total number of students (mean, SD)	596.0 (120.9)

School characteristics were obtained from Texas Academic Performance Reports from the Texas Education Agency (https://tea.texas.gov/).

**Table 3:** Physical activity benefits for students discussed by school staff.

Academic	Makes students more engaged, helps learning, provides academic break, improves comprehension, improves focus, improves ability to think, stimulates learning, helps get students ready to learn, motivates learning, improves recall of information, improves test scores, improves attention span, helps students learn about sports, improves attendance, helps reading, helps math, improves memory, improves on task behavior, improves ability to concentrate
Social-emotional	Improves mood, improves teamwork, provides hope, decreases disciplinary issues, improves behavior, helps students decompress, helps mental health, calms students down, helps relax students, builds confidence, improves class dynamics, reduces stress, allows students to gain new experiences, improves school enjoyment, improves camaraderie, improves sportsmanship, helps students make new friends, provides a coping mechanism, improves social behaviors, improves school engagement, develops life skills, decreases peer conflict, builds communication skills, helps students learn the art of play, promotes team work, promotes cooperation
Physical	Makes students more alert, improves weight management, improves prediabetes, brings oxygen to the brain, helps physical development, helps movement skills, improves energy levels, supports brain development, stimulates the brain, improves cardiovascular health, helps brain health, fosters skill development, improves ability to sit longer, improves motor skills, improves athletic skills
Instructional	Improves quality of classroom time, builds relationships between students and teachers, allows for more teaching time, reduces interruptions for behavior management during teaching, helpful instructional tool, helpful during class transitions

### Academic benefits

#### Learning readiness

Participants commonly discussed how students were more ready to learn after physical activity because they were able to sit longer and pay attention. An assistant principal (participant 5) stated: “When the kids come back from those labs [motorlabs], they're much more ready, more ready to learn, and pay attention and sit down and concentrate.” A teacher (participant 11) also explained: “As teachers come in and use the motorlab and bring their class and they do really see the benefits of having those stations and having them have brain breaks and…having that stimulation and then going back into the classroom and just being ready to learn.”

Teachers also observed learning readiness improve after recess and physical education. A teacher (participant 9) described her students after recess: “…they're running, they're playing, and they come back and they refresh. Then they're ready to learn.” She continued to explain how they performed academically: “They come back. They get their work right away. They go in within five minutes. They bring it back to me, and it's all correct.” Another teacher (participant 6) described how her students performed better on physical education days: “You can tell the days that are health fitness days, and the days that aren't. Their behavior, their attention span, their energy levels are better.”

#### Learning engagement

Participants also discussed that students enjoy physical activity opportunities and movement, which engaged them in learning. Providing students with movement was thought to be a motivator for learning. A PE teacher (participant 4) explained how her school was working through a redesign process, with feedback from children. This process revealed that students wanted more movement at school: “They [the school] interviewed over 100 kids and they all said they're bored. They're bored, they don't move, they need more movement, they want to play more.” The PE teacher (participant 4) further explained that: “The more movement they [students] get, the more bought in to school they are, then—the more they come to school, the more they love school. I think our struggling kids—if you have something that motivates them, then they want to do well for you, they want to behave for you.”

Participants also explained how students were more engaged learners when movement was incorporated into academic lessons. For example, a classroom teacher (participant 8) highlighted: “If you give them [students] physical activities, then they will want to learn more.” A principal (participant 10) also explained how students like to move and benefit when teachers use lessons that include movement: “They [students] want to get up and move around. So, they [teachers] can reinforce those same literacy skills in that lab [motorlab] with the kids moving and they're going to probably get a lot more out of those thirty minutes than they would just sitting down doing another reading group.”

#### Academic performance

Participants discussed how integrating movement into lessons directly improved academic performance. For example, a PE teacher (participant 4) integrated sight words and math facts into motorlab stations and described her experience: “I did a pre-assessment of sight words and math facts for the kids…and then the second assessment after going through the stations and practicing the same sight words and the same math facts but adding movement to it, then I did a post-assessment. I would say ninety-three percent of our kids show progress right away, and then the students that show little progress, after another two days in the lab they're showing extreme amounts of progress.”

A classroom teacher (participant 8) explained how student spelling performance improved when teaching with movement compared to using an oral/auditory approach. She highlighted how student's recall was better because they associated a word with a movement: “…they're able to recall because they have that movement with the information that they've learned. So, I've seen like—if I just do a straight spelling test, and I'm verbally telling them, and then they do the spelling test, and it's memorize it. They might do well, but they're not going to apply it. So maybe later on I can see, ‘Oh, you know, remember that we were doing this; we call Spelling Ladders? You're doing the Spelling Ladders.’ And they'll spell those words a little bit better than they would—just straight oral or audio learning.”

### Social-emotional benefits

#### Behavior

School staff perceived that providing physical activity opportunities helped improve students' behavior and reduce disciplinary issues. A teacher (participant 9) explained that students have a natural need to move, and that physical activity opportunities alleviate the uncontrolled fidgeting that led to students getting in trouble: “I see kids fidgeting and moving around and getting in trouble, and it's not up to them. They have no control over it. And I feel like if they were able to go outside and play, a lot of that would be alleviated.” Likewise, an assistant principal (participant 3) explained how providing physical activity opportunities served as an outlet for students: “I'm in charge of discipline. And one of the things that I do is I'll go get kids, and we'll go to the gym, and they'll run, or we'll walk the track, or things like that because knowing the students, that outlet is something that helps them.” Another assistant principal (participant 15) noted how the lack of movement opportunities can lead to conflicts for students: “There's not a whole lot of opportunity for them to move about the classroom. And so, I feel like the students get tired. Some of them are a little more fidgety than others. They don't really have those opportunities for them to move about. And so that can result in different conflicts, whether with peers, with their teachers.”

#### Interpersonal/social skills and classroom culture

Participants described how physical activity opportunities helped improve student's interpersonal skills, their social skills, and class culture. For example, an assistant principal (participant 5) explained: “It [recess] builds the communication skills between the kids. It builds their sense of union with the class, and then they also need to move throughout the day.” Likewise, a PE teacher (participant 2) commented that: “The benefits (of PE) are for one, enjoyment, the camaraderie…We teach sportsmanship… Here in health fitness, they don't get to always choose their teams, so they are engaged in games and activities with the friend or they make a new friend, they find out how to make new friends.” A classroom teacher (participant 6) further reinforced how PE and the motorlab helped build self-confidence and improve group dynamics: “my class loves health fitness—can't wait for it to be health fitness, especially motorlab day. It builds their confidence. It makes the team dynamics and the group dynamics stronger.”

Another assistant principal (participant 3) explained how structured activities help build important relationship and teamwork skills: “I also think they learn just the art of play and interacting with each other and taking turns and working as a team and collaboration and cooperation.” She (participant 3) noted that this is particularly important for students who lack opportunities for sport outside of school, due to lower socio-economic status: “And prior to our partnership with the community center, we had forty percent of the kids who did not participate in anything outside of school in regards to athletics…For me, the idea of organized sports is always a positive thing for kids. I mean, you're learning not only the athletic skills, but the socialization piece, the collaboration, and the teamwork. And I don't think we have things for all of our kids in that regard.” This theme was reinforced in the subtheme of motor/sport skill development.

### Physical health benefits

#### Brain health

When discussing benefits of physical activity many participants referred to existing research about the impact of physical activity on the brain. For example, a principal (participant 16) cited heat maps of brain activity after physical activity: “…physical activity, I believe, helps the brain kind of become more alert, and kind of wakes you up a little bit more. And I think it's beneficial for learning as opposed to being sedentary…I see the image of the red, the redness. You know, when it [the brain] looks like it's alert, you know, after physical activity.” A PE teacher (participant 1) also described a graphic comparing brains after sitting versus 20 min of walking: “There's a nice graphic on here's what your brain looks like, and here's your brain on twenty minutes of walking, and just seeing the activity in your brain, which is a really good graphic.” Participants also connected the brain research to general health and learning. For example, a principal (participant 10) stated: “There's just so much brain research that backs movement and learning.” Later, she stated: “In order for your brain to be healthy and well, your body has to be healthy and well. So, I feel like the two go hand in hand. You cannot have success academically without movement and without structured opportunities to play and run.”

#### Motor/sport skill development

A principal (participant 12) explained how it is important for schools to help students develop movement and athletic skills at a young age. This is especially important for students who lacked the resources to participate in physical activity outside of school (e.g., many of the students at Title 1 schools). Otherwise, students may not get opportunities to build skills that can serve them as they get older: “If kids aren't trained how to play soccer, use a hula hoop, anything, play jump rope, and again our kids don't even have some of those basic skills just because they don't have a place to do it after school…there's no park around here that you would see that.” Likewise, a teacher (participant 6) explained: “A lot of them don't get to play Little League or attend the YMCA. It's not until seventh grade where they actually see sports. So, I think our health fitness has tried to bring those up—last year they went for a baseball throwing, and the kids just lit up. ‘I can't throw a baseball.’ And I'm like, ‘You're six years old. You've never thrown a baseball?’ Like it just breaks my heart…” Similarly, a PE teacher (participant 14) noted: “…For kids that were apartment kids, this was their outside activity, being at school. This was when they got to be out and free, not worry about trying to play in a parking lot or—they could come to school, and we had our structured classes, and they were able to learn new sports.”

#### Physical health and chronic conditions

While participants also discussed physical health benefits, these were not as commonly discussed, or they were mentioned among other benefits. For example, a principal (principal 13) explained how sports can help build athletic skills, teamwork skills, and address health issues. “Our kids, they live in apartments. Their parents work late. And so, they need somewhere to go. And again, I think them learning to play sports, baseball, soccer, any kind of support, helps to give them that sense of participation, teamwork. And then, again, just to also keep them active, you know, physically healthy.” When discussing brain breaks, a teacher (participant 8) acknowledged the cardiovascular benefit in addition to other benefits: “It's not just cardiovascular. It's also how to decompress, how to have fun with movement and songs…”

### Instructional benefits

#### Quality teaching time

Participants reported multiple instructional benefits for teachers when providing students with physical activity opportunities. One benefit was more efficient and quality teaching time. A teacher (Participant 6) explained how there were fewer redirections and less time spent managing behavior or reteaching content if students had recess breaks: “If you think about all the wasted time that they [teachers] have for behavior management or kids not focused…I think if teachers just gave up a little bit of the control and structure and said, ‘Okay, we are going to do three recesses,’ because once the kids get back from those recesses they're focused…they can really learn instead of wasting the fifteen minutes redirecting or having to reteach because the kids aren't focused…I think teachers and even parents might say, ‘Well, it's a big waste of time,’ but I think if it's done correctly, it's not, it's a huge gain.” A principal (participant 16) shared similar views: “You're going to get more out of them—when you give them that little break, and you give them that time.” A teacher (participant 8) attributed more teaching time to greater engagement: “And so, physical activity allows me to have more teaching time because they'll [students] buy into it.”

#### Teaching and class management tool

Participants viewed classroom-based physical activity approaches as an instructional tool that could aid in teaching academic content and support classroom management. For example, a principal (participant 12) explained: “I feel like the physical activity is almost like an instructional tool that could help them to reach those academic goals.” A PE teacher (participant 4) also described how brain breaks were a helpful way for teachers to transition between classes: “Some of our teachers use it for transitioning to different classes. So, before they come to specials or after they come to specials, they do a brain break to get them focused.” Another principal (participant 10) explained how physically active breaks were helpful for classroom management: “Or in the middle of class, if the teacher notices she's giving her lesson and the kids are just not focusing, let's just stop, stand up touch your toes, take a deep breath, and sit down and then she just gets right on with her lesson. So, it's really a tool for them to use as needed throughout the day.”

#### Teacher-student relationships

Teachers also reported how physical activity opportunities can improve their relationships with students. For example, a teacher (participant 6) described how playing with students during recess improved their relationships: “They love when I have recess duty because I'll go out there and play with them…that part of that relationship with the kids is really big for me. It just sets a different tone in a relationship building where these kids—a lot of them don't have very much.” When discussing the benefits of a structured recess program, a PE teacher (participant 4) acknowledged how it helped build teacher-student relationships: “I think that was a huge benefit to our kids because they had specific games and areas and zones that they wanted to play in. The teachers also were required to participate with them, so building their relationships, and then they had a lot of student ownerships.” Another teacher (participant 8) described the benefit: “But I always felt it's worth that five minutes because then you get more work out of the children because their blood is flowing, their heart is pumping, and they're like, ‘Wow, this is—you are like the coolest teacher ever who is letting us do this.’ That's when you know you're doing well is when the kids keep going.”

## Discussion

Our findings highlight multiple benefits teachers and staff observed or perceived when providing students with physical activity opportunities during school. Staff directly observed how students enjoyed learning through movement and had a natural desire to move. Students were also more ready to learn, more engaged, and better behaved. Additionally, physical activity opportunities were ways to build interpersonal and social skills and contributed to a positive classroom culture. Teachers and staff also discussed physical health benefits by emphasizing research about physical activity and brain health, and noted the importance of physical activity for skill development, especially at schools serving economically disadvantaged students who otherwise lacked access outside of schools. There were also reported instructional benefits for teachers, which included more quality teaching time, tools to improve lessons and classroom management, and improved relationships with their students.

Numerous studies have examined many of the observed/reported benefits of physical activity for students, providing empirical support for teacher and staff perceptions found in our study. For example, studies have reported beneficial effects (or positive associations) of physical activity on key academic-related outcomes including: attention (4), time-on-task (7, 8, 31), school engagement (32), learning behavior (33), math performance (3), academic performance (4), academic achievement (5), and working memory (4). Additionally, studies have reported the value of integrating movement into learning activities with specific academic goals, such as learning vocabulary (34, 35). Studies have also reported benefits of physical activity for social-emotional outcomes such as student behavior (9), self-regulation (6), and internalizing and externalizing behavior (10). Further, studies have reported physical activity benefits for the participant described physical health-related outcomes such as “brain health” (i.e., brain activity, executive function, and cognitive function) (4, 5, 36), motor skills (9, 37, 38), obesity (39), and cardiovascular health (40). Fewer studies have examined the impact of physical activity on social skills, classroom culture, or the reported instructional benefits, which may represent areas of future work.

Despite the observed benefits of physical activity for learning (and other key behavioral outcomes), and existing empirical research to support many of these benefits, physical activity is often viewed as a lower priority in schools (19, 20). For example, competing priorities and lack of time are two commonly reported barriers to implementing physical activity approaches (19). Our findings suggest that physical activity opportunities may be strategically integrated within the school day to enhance learning for students rather than compete with it. For example, teachers may use brain breaks at strategic times to help improve learning readiness or plan physically active lessons to further engage students in learning. School administrators may also structure the school schedule to include daily recess and regular physical education at strategic times so students have structured and unstructured movement opportunities built in to their days. These approaches can be helpful given staff reported that when student's movement needs were met, they were more engaged and focused, rather than being disruptive and requiring teachers to manage behavior and repeat lesson components.

In addition to lack of time and competing priorities, there are other reported barriers to implementing physical activity opportunities in school. Specifically, a lack of resources, unsupportive cultures, a lack of leadership support, limited staff capacity, and a lack of staff buy-in can all negatively impact implementation efforts (18–21). Our findings can further inform how public health professionals and educators communicate how physical activity can support academics, behavior, social-emotional well-being, and teacher-student relationships. Effective communication strategies can help address common implementation barriers by shifting leader's and staff's perspectives about the role of physical activity. Engaging leaders is an important first step to establishing school cultures that are supportive of movement, and attaining more resources for staff implementers. Effective communication with staff can help improve buy-in.

There are several key implications from our findings. First, our results provide guidance for researchers and practitioners for engaging with school leaders, teachers, and staff about the importance of physical activity in schools. Advocates should emphasize how physical activity can improve learning readiness, classroom engagement, and academic performance, and thus directly aligns with academic goals rather than competes with them. Additionally, messaging should highlight how teachers can have more effective and efficient classroom time and may actually get time back if movement opportunities are provided in strategic and purposeful ways. These two messages address two commonly reported barriers to physical activity promotion in schools (lack of time and competing priorities). Multiple school staff members also referenced the images of the brain after 20 min of sitting versus walking (36), indicating the usefulness of incorporating key images from research studies when discussing student benefits of engaging in physical activity.

Another implication is that public health and wellness advocates need to engage academic and student behavior experts in physical activity promotion efforts in schools. Physical activity should not be limited to a physical education or wellness department. Instead, it should be considered part of a whole-of-school approach and integrated throughout curriculum and instruction, classroom management strategies, and school culture. Shifting the narrative for school personnel in this way has the potential to engage a diverse group of leaders in a school system who have the decision-making power to ensure schools provide physical activity opportunities to students.

A third implication is our results can inform additional outcomes and goals of physical activity promotion to consider when conducting school-based physical activity research. In addition to the academic and social-emotional outcomes, our findings highlight ways in which physical activity opportunities can improve interpersonal skills, social skills, classroom culture, and teacher-student relationships. Interpersonal skills may be improved through cooperative games and lessons that require students to positively interact with their teachers and peers. Additionally, educators that leverage movement-based activities and lessons have the potential to improve student focus and reduce negative behaviors, leading to a more positive classroom environment and better relationships among students and teachers. These outcomes are of high importance as they have the potential to improve the broader school culture, provide students with valuable life-long skills, and address disparities. However, more empirical research is needed to determine the extent to which these outcomes may be impacted by the various types of physical activity opportunities that schools provide.

### Limitations/strengths

This study has limitations. The recruitment approach may have favored those with positive views about physical activity and the corresponding benefits. The research team intentionally tried to recruit participants regardless of their views about physical activity, but those with more negative views may have been less willing to participate given the study was about physical activity. Further, we did not collect background information from interviewees about their previous trainings and experiences with physical activity. This would have been valuable information to better understand the sample and what may shape their perspectives. Additionally, interview participants were from a single school district. Although the district did serve many schools, the ways in which the district broadly promoted physical activity (e.g., through trainings and materials provided) could have shaped participants' experiences and perceived benefits. We did not collect data from parents and students, for which their views could have added valuable information to this study. Lastly, qualitative data were collected prior to the COVID-19 pandemic, so perspectives may have since shifted given the many intended and unintended consequences of the pandemic on school systems and physical activity opportunities provided in schools (41, 42).

There are several strengths. We obtained multiple perspectives about physical activity benefits by enrolling staff members from different job types. We also found consistency across job types reinforcing findings about observed and perceived benefits. We also engaged the district wellness team throughout the study as they reviewed our interview guide, helped with recruitment, and reviewed preliminary findings. The partner district and schools were using multiple approaches to support student physical activity. Thus, participants were able to speak to a wide range of physical activity opportunities and their corresponding benefits. This study provides a rich understanding of potential physical activity benefits in schools, which can stimulate future studies to explore in other samples and further examine through experimental designs.

## Conclusions

Physical activity has been traditionally viewed as a secondary priority in schools, limiting the scalability, implementation, and effectiveness of school-based programming. Our study highlights ways in which educators directly observed how physical activity can improve academic and social-emotional outcomes for students. Given the empirical research supporting many of these observed benefits, there is a need to shift the narrative about physical activity in the school setting from a supplemental programming perspective, to one in which physical activity programming is viewed as an integrative and/or synergistic approach. Physical activity plays a critical role in schools and can directly contribute to their educational success. Physical activity also has the potential to enhance students' social skills, positively impact classroom culture, and improve the quality and efficiency of classroom teaching time. Researchers should continue working with schools to improve the breadth and depth of school-based physical activity approaches and strive to create lasting partnerships with school personnel, as results from our study indicate our priorities are indeed aligned.

## Data Availability

The datasets presented in this article are not readily available. For privacy protection, we do not plan to share the original data. Requests to access the datasets should be directed to TW, timothy.j.walker@uth.tmc.edu.
